# Combined Pharmacological Induction of Hsp70 Suppresses Prion Protein Neurotoxicity in Drosophila

**DOI:** 10.1371/journal.pone.0088522

**Published:** 2014-02-11

**Authors:** Yan Zhang, Sergio Casas-Tinto, Diego E. Rincon-Limas, Pedro Fernandez-Funez

**Affiliations:** 1 Department of Neurology, McKnight Brain Institute, University of Florida, Gainesville, Florida, United States of America; 2 Department of Molecular, Cellular and developmental Neurobiology, Instituto Cajal, Madrid, Spain; 3 Department of Neurosciences, Genetics Institute, and Center for Translational Research on Neurodegenerative Diseases, Gainesville, Florida, United States of America; 4 Center for Movement Disorders and Neurorestoration, Gainesville, Florida, United States of America; University of Maryland School of Medicine, United States of America

## Abstract

Prion diseases are rare and aggressive neurodegenerative disorders caused by the accumulation of misfolded, toxic conformations of the prion protein (PrP). Therapeutic strategies directed at reducing the levels of PrP offer the best chance of delaying or halting disease progression. The challenge, though, is to define pharmacologic targets that result in reduced PrP levels. We previously reported that expression of wild type hamster PrP in flies induces progressive locomotor dysfunction and accumulation of pathogenic PrP conformations, while co-expression of human Hsp70 delayed these changes. To validate the therapeutic potential of Hsp70, we treated flies with drugs known to induce Hsp70 expression, including the Hsp90 inhibitor 17-DMAG and the glucocorticoid dexamethasone. Although the individual treatment with these compounds produced no significant benefits, their combination significantly increased the level of inducible Hsp70, decreased the level of total PrP, reduced the accumulation of pathogenic PrP conformers, and improved locomotor activity. Thus, the combined action of two pharmacological activators of Hsp70 with distinct targets results in sustained high levels of inducible Hsp70 with improved behavioral output. These findings can have important therapeutic applications for the devastating prion diseases and other related proteinopathies.

## Introduction

Prion diseases encompass a diverse group of rare, aggressive, and incurable neurodegenerative conditions characterized by spongiform brain degeneration and accumulation of insoluble isoforms of the prion protein (PrP) [1]. Creutzfeldt-Jacob disease (CJD) is the most common prion disease in humans and typically presents with cognitive perturbations that may overlap with other dementias, but it has an unmistakable short course following diagnosis. Other forms of prion diseases include Gerstmann-Straussler-Scheinker syndrome, kuru, and fatal insomnia, which can present with cognitive, behavioral, and/or locomotor disturbances [1]. These distinct disorders are caused by the accumulation of aberrant, toxic conformations of the PrP, a membrane-anchored glycoprotein widely expressed in the brain [2]. PrP has two main domains, an unstructured N-terminus and a globular C-terminus with three α-helices and two short ß-strands [3], [4]. Rearrangements of the globular domain that increase ß-strand content at the expense of α-helices are proposed to mediate the conversion of PrP into pathogenic conformations. These structural perturbations induce prominent changes in the biochemical properties of PrP, including insolubility, aggregation, and, in most cases, resistance to denaturing agents and proteinase K (PK) [2]. However, the mechanisms regulating the conformational changes of PrP are still poorly understood. What is clear, though, is that misfolded PrP isoforms trigger neurodegeneration and, thus, strategies directed at reducing the levels of abnormal PrP should have therapeutic benefits. Unfortunately, no clinical treatments with demonstrated benefits against prion diseases exist at this time.

Using a *Drosophila* model of prionopathies, we previously showed that wild type PrP from Syrian golden hamster induces progressive spongiform brain degeneration, locomotor dysfunction, and accumulation of pathogenic PrP conformations [5]. Interestingly, co-expression of the Heat shock protein 70 (Hsp70), a molecular chaperone that prevents and reverts protein misfolding, reduced the levels of total PrP, prevented PrP misfolding, and was neuroprotective. We also showed that Hsp70 was present in the lipid raft domains in which PrP is enriched and interacted directly with PrP, thus suggesting that Hsp70 can directly regulate PrP misfolding and promote its degradation [5]. We later showed that recombinant Hsp70 inhibits PrP conversion in vitro [6], further supporting the ability of Hsp70 to prevent the accumulation of transmissible PrP conformations. Interestingly, Hsp70 directly interacts with cytosolic PrP in cultured cells, displacing Bcl-2 from insoluble Bcl-2/cytosolic PrP complexes, thus liberating soluble Bcl-2 to prevent apoptosis [7]. Other indirect evidence further links Hsp70 to prion diseases. For instance, expression of Hsp70 increases several fold in mice infected with scrapie [8] as well as in CJD patients [9], [10]. This observations indicate that PrP misfolding induces a protective stress response in the early stages of disease, a phenomenon observed in other protein misfolding disorders (reviewed in [11]). This proteotoxic stress response may be responsible for the typical late onset of most proteinopathies, suggesting that the protective mechanisms are overwhelmed after several years of protein accumulation, leading to neuronal dysfunction and cell loss. Overall, this evidence suggests that Hsp70 could be a valuable therapeutic target in prion diseases and other related proteinopathies.

Heat shock proteins (HSPs) are a class of highly conserved molecular chaperones that facilitate protein folding and prevent protein aggregation. They also play essential roles in other cellular processes, including protein targeting, transport and translocation, autophagy, proteasomal degradation, and signal transduction [11], [12]. Hsp70 and Hsp90 are two functionally related ATP-dependent chaperones that have become relevant therapeutic targets in cancer and neurodegeneration in the last few years. Hsp90 is a ubiquitous chaperone implicated in a number of signaling cascades [13] and in the stabilization of nuclear hormone receptors [14]. Furthermore, *in vitro* studies have shown that Hsp90 negatively regulates stress pathways by binding to and inhibiting its central transcriptional mediator, Heat shock factor 1 (HSF1) [15], [16]. HSF1 regulates the transcription of various HSPs by directly interacting with specific binding sites in their regulatory region during cellular differentiation or stress. One transcriptional target of HSF1 is the family of inducible Hsp70, the main cellular chaperone in animals, thus functionally linking Hsp90 and Hsp70. Although Hsp90 and Hsp70 cooperate to prevent protein aggregation, Hsp90 negatively regulates the expression of inducible Hsp70, resulting in a feedback loop that limits the response to stress.

Geldanamycin is a naturally occurring ansamycin antibiotic with demonstrated anti-cancer activity, particularly in tumors associated with abnormally elevated levels of receptor tyrosine kinase activity [13]. Subsequent studies showed that geldanamycin binds the ATPase domain of Hsp90 and inhibits its ATP-dependent activity [17]. In addition to this anti-cancer activity, the N. Bonini lab showed that geldanamycin was an effective Hsp70 inducer that protected flies against α-synuclein toxicity [18], although later studies showed that similar benefits could be obtained with low doses of geldanamycin that do not induce Hsp70 expression [19]. Other groups have reported similar benefits of geldanamycin in fly and mouse models of Huntington's disease [20], [21], supporting the benefits of this compound in protein misfolding disorders. Despite these positive results, geldanamycin presents important limitations as a therapeutic agent. Geldanamycin works in the micromolar range and is water insoluble, two undesirable properties for clinical application. Additionally, geldanamycin has demonstrated high hepatic toxicity in animal, limiting its suitability for clinical use [22]. Fortunately, extensive research to produce geldanamycin derivatives with better pharmacodynamics resulted in the discovery of a small family of functional derivatives, including 17-allylamino-17-demethoxy-geldanamycin (17-AAG), the first analogue to progress to clinical trials, and 17-dyimethylaminoethylamino-17-demethoxygeldanamycin (17-DMAG), the first water-soluble analogue of geldanamycin, which provides improved bioavailability and tissue distribution [23]. 17-AAG also suppressed neurodegeneration in *Drosophila* two models of polyglutamine diseases [24]. A recent assessment of these geldanamycin analogues indicates that they were halted in phase 1/2 clinical trials due to several concern, including poor bioavailability (17-AAG) and toxicity (17-DMAG) [25].

In addition to the geldanamycin family of Hsp90 inhibitors, it may be useful to identify other compounds that can induce Hsp70 expression by other mechanisms. Dexamethasone is an FDA-approved glucocorticoid with multiple beneficial activities. The best-known clinical application of dexamethasone is as an anti-inflammatory and immunosuppressant. Among its many proposed activities, dexamethasone has been proposed to directly activate HSF1. Treatment of rat cardiac myocytes with 10 and 100 µM dexamethasone increased HSF1 stability on its binding sites, resulting in the selective induction of Hsp70 expression, but not Hsp27 or Hsp60 [26]. Additionally, the combination of L-asparaginase and dexamethasone, but not the corticoid prednisone, induced the expression of several HSPs, resulting in reduced risk of thrombosis in patients with acute lymphoblastic leukemia [27]. There is also evidence that Hsp90 and other HSPs interact with the glucocorticoid receptor and that binding to steroid hormones or glucocorticoids causes the dissociation of this complex, liberating HSPs for other functions, including protein dyshomeostasis [28].

Following up on our preliminary observations that Hsp70 misexpression protects against PrP neurotoxicity, here we examined the ability of Hsp70-inducing compounds to mitigate PrP misfolding and neurotoxicity in flies. Although the individual treatments with 17-DMAG and dexamethasone produced no significant benefits, a combined regimen of 17-DMAG and dexamethasone reduced the steady-state levels of PrP, decreased the levels of pathogenic PrP conformers, and improved the locomotor performance of the flies. These results indicate that pharmacological induction of Hsp70 activity promotes PrP degradation and prevents PrP misfolding. Therefore, we have identified a new target for the therapeutic treatment of prion diseases – HSF1 – and a combination of drugs with a potent stimulatory effect on the HSF1 transcriptional target Hsp70.

## Results

### 17-DMAG does not affect PrP expression

The purpose of these studies was to demonstrate that pharmacological induction of Hsp70 could achieve the same beneficial effects against PrP accumulation and neurotoxicity that we observed with Hsp70 misexpression [5]. As our main assay, we analyzed the steady-state levels of PrP in day 1 flies, which were significantly reduced by Hsp70 misexpression in our previous study [5]. To study PrP expression, we raised flies expressing moderate levels of PrP under the control of the weak, ubiquitous driver da-Gal4. To induce Hsp70 pharmacologically, we replicated published studies of flies treated with geldanamycin with concentrations up to 96 µg/ml [18]. However, we experienced high toxicity when feeding flies with geldanamycin at 24 and 48 µg/ml, and this toxicity was highly dependent on manufacturer and batch, which precluded the consistent use of the optimum concentration of 96 µg/ml geldanamycin published before [18].

**Figure 1:**
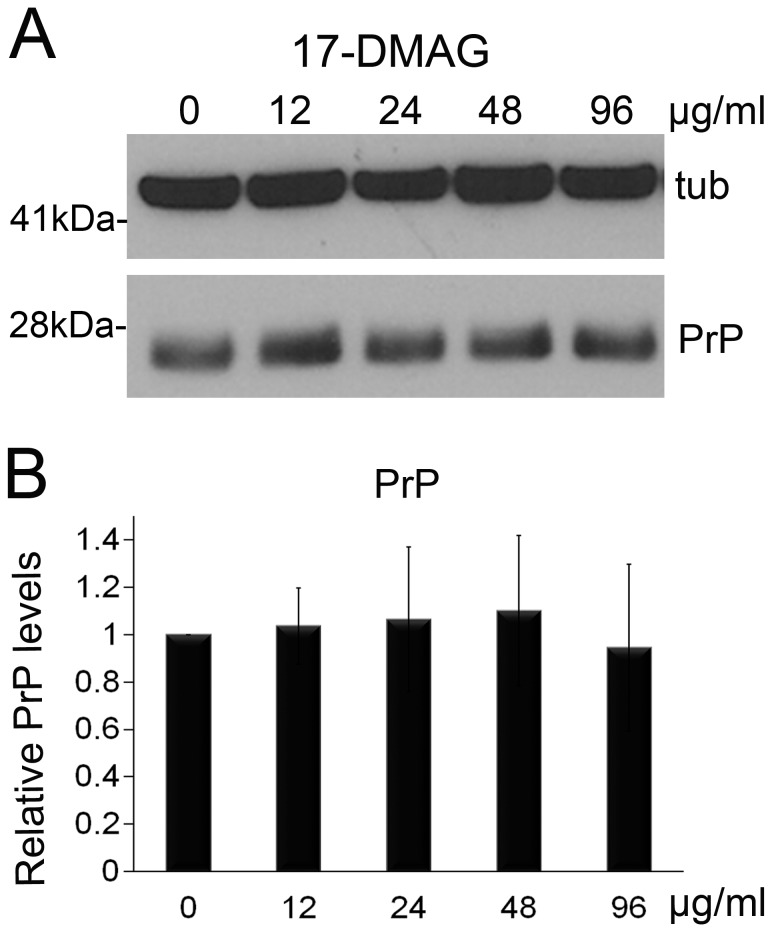
17-DMAG has no effect on PrP expression. **A**, Flies expressing PrP ubiquitously (da>PrP-M9) were fed with 17-DMAG at 0, 12, 24, 48, and 96 µg/ml during development. Analysis of PrP levels in 1 day-old flies by western blot showed no significant changes. Tubulin was used as loading control to normalize protein levels. **B**, Quantification of the triplicate experiment followed by analysis with ANOVA. No statistical differences were identified in these experiments.

Fortunately, a few derivatives of geldanamycin recently become commercially available. Several of these analogues work at sub-micromolar concentrations, but are poorly soluble in water, like 17-AAG. In contrast, 17-DMAG is water soluble, making this compound the best candidate for in vivo delivery [23]. Taking advantage of its solubility, we incorporated 17-DMAG at concentrations between 12 and 96 µg/ml into standard fly media to make it available throughout all developmental stages to flies expressing PrP. Then, we collected 1 day-old flies and conducted western blot to compare the steady-state levels of total PrP using the 6D11 anti-PrP antibody. For quantification, we performed the experiments in triplicate and detected tubulin as loading control to normalize expression data. After averaging the normalized PrP levels, we found that PrP expression was mostly unchanged in the 17-DMAG treatments (Fig. 1A and B and Table 1).

**Table 1:** Statistical comparisons (ANOVA) for PrP levels in flies treated with 17-DMAG.

Test details	Mean 1	Mean 2	Mean Diff.	P value Bonferroni's test	P value Dunnett's test	Difference/significant
12 µg/ml v non-treatment	1.036	1	0.03555	>0.9999	0.9951	NS
24 µg/ml v non-treatment	1.065	1	0.06496	>0.9999	0.9572	NS
48 µg/ml v non-treatment	1.101	1	0.1014	>0.9999	0.8373	NS
96 µg/ml v non-treatment	0.9448	1	−0.0552	>0.9999	0.9754	NS

### Dexamethasone does not affect PrP expression

**Figure 2:**
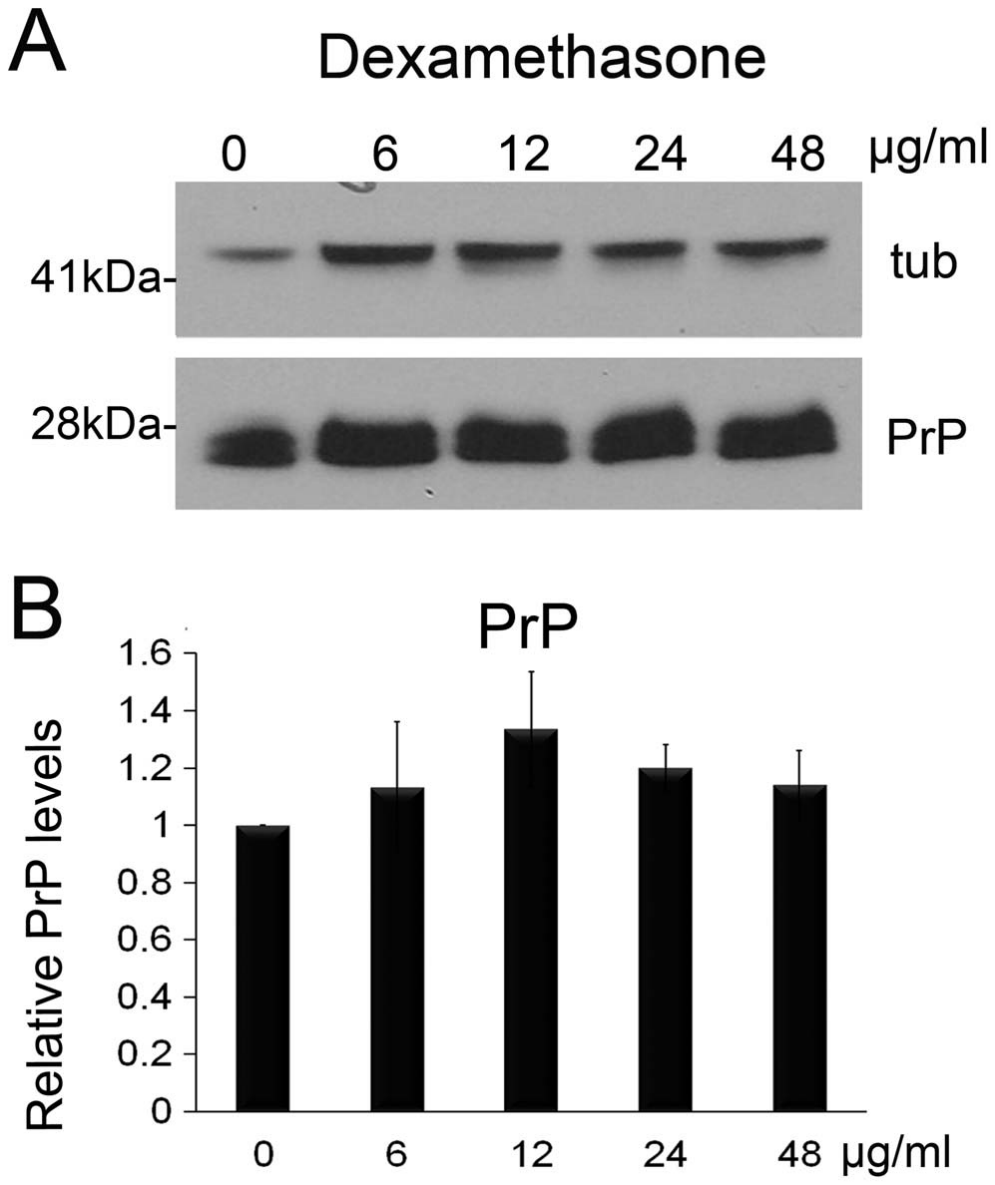
Dexamethasone has no effect on PrP expression. **A**, Flies expressing PrP ubiquitously (da>PrP-M9) were fed with 17-DMAG at 0, 6, 12, 24, and 48 µg/ml during development. Analysis of PrP levels in 1 day-old flies by western blot showed no significant changes. Tubulin was used as loading control to normalize protein levels. **B**, Quantification of triplicate experiment followed by analysis with ANOVA. No statistical differences were identified in these experiments.

An alternative to using higher concentration of geldanamycin derivatives was to identify a second drug that could reinforce the activity of 17-DMAG. A literature search identified dexamethasone as an FDA-approved glucocorticoid that induces Hsp70 by promoting the stability of HSF1 on the Hsp70 promoter [26]. To examine the effect of dexamethasone on PrP accumulation, we raised flies expressing PrP ubiquitously as described above in fly media containing dexamethasone at concentrations from 6 to 48 µg/ml. Then, we collected 1 day-old flies, performed western blot, quantified the bands, and normalized the expression with tubulin to compare the three independent experiments. Overall, no dexamethasone treatment produced significant differences (Fig. 2 and Table 2). Although the 12 µg/ml treatment showed 30% higher levels of PrP, this difference was not significant (*p* = 0.07). Thus, neither 17-DMAG nor dexamethasone had significant effects on PrP steady-state levels when used alone.

**Table 2:** Statistical comparisons (ANOVA) for PrP levels in flies treated with dexamethasone.

Test details	Mean 1	Mean 2	Mean Diff.	P value Bonferroni's test	P value Dunnett's test	Difference/significant
6 µg/ml v non-treatment	1.132	1	0.1315	>0.9999	0.6666	NS
12 µg/ml v non-treatment	1.337	1	0.3369	0.0951	0.0709	NS
24 µg/ml v non-treatment	1.199	1	0.199	0.5551	0.3545	NS
48 µg/ml v non-treatment	1.142	1	0.1417	>0.9999	0.6144	NS

### Combined treatment of 17-DMAG and dexamethasone reduces PrP levels

**Figure 3:**
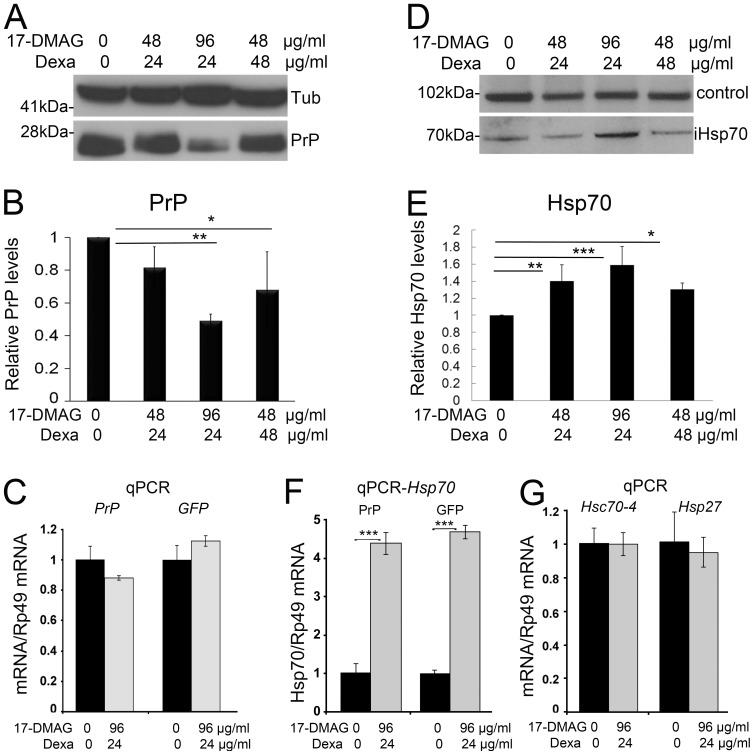
Co-treatment of 17-DMAG and dexamethasone reduces PrP and induces Hsp70. **A** and **B**, Flies expressing PrP ubiquitously (da>PrP-M9) were fed with combinations of 17-DMAG at 0, 48, and 96 µg/ml and dexamethasone at 0, 24, and 48 µg/ml during development. Analysis of PrP levels in 1 day-old flies by western blot showed significant reduction in PrP in two combinations following quantification and analysis by ANOVA, with 17-DMAG 96 µg/ml and dexamethasone 24 µg/ml inducing the strongest response. **C**, Analysis of *PrP* and *GFP* mRNA by qPCR showed no significant changes in flies treated with the optimum drug combination. **D** and **E**, Western blot and quantification of Hsp70 in flies treated with different combinations of 17-DMAG and dexamethasone. Quantification of Hsp70 followed by normalization with a non-specific band (control) revealed that all treatments induced significant increases in Hsp70, with 17-DMAG 96 µg/ml and dexamethasone 24 µg/ml inducing the strongest response. **F**, Expression of *Hsp70* mRNAs by qPCR revealed a four-fold increase in flies treated with the optimum 17-DMAG/dexamethasone cocktail in flies expressing either PrP or CD8-GFP. **G**, Analysis of *Hsc70-4* and *Hsp27* mRNA by qPCR in flies treated with the optimum 17-DMAG/dexamethasone cocktail showed no significant changes in these two chaperones. Statistical significance was analyzed by ANOVA multiple comparisons for western blots and t-test for qPCR: * *p*<0.05; ** *p*<0.01; *** *p*<0.001.

Since 17-DMAG and dexamethasone have different molecular targets, but result in Hsp70 induction, these compounds offer the opportunity to examine their potential synergistic activity. To explore the potential benefit of a combined regimen of these two Hsp70-inducing compounds, we raised flies expressing PrP ubiquitously as above in standard media containing combinations of high concentrations of 17-DMAG and dexamethasone or control media (Fig. 3A). Then, we collected 1 day-old flies, performed western blot, and quantified the bands as described above. All drug combinations tested reduced total PrP compared with non-treated controls, although only two conditions were significantly reduced (Figs. 3A and B). The combinations of 17-DMAG at 48 µg/ml with dexamethasone at 24 µg/ml resulted in 19% reduction in total PrP, but did not reach statistical significance (*p* = 0.2) (Table 3). The combination of 96 µg/ml of 17-DMAG and 24 µg/ml of dexamethasone produced a 51% reduction in the steady-state levels of PrP that was statistically significant (*p* = 0.0036). Also, 48 µg/ml of 17-DMAG and 48 µg/ml of dexamethasone reduced PrP by 32% (*p* = 0.03). Thus, the combined treatment with 17-DMAG and dexamethasone achieved a robust reduction of PrP stead-state levels.

**Table 3:** Statistical comparisons (ANOVA) for PrP levels in flies treated with combinations of 17 DMAG and dexamethasone.

Test details	Mean 1	Mean 2	Mean Diff.	P value Bonferroni's test	P value Dunnett's test	Difference/significant
48+24 v non-treatment	0.8169	1	−0.1831	0.2752	0.202	NS
96+24 v non-treatment	0.4922	1	−0.5078	0.0043	0.0036	**
48+48 v non-treatment	0.6803	1	−0.3197	0.0384	0.0307	*

* ***p*** **<0.05;**

** ***p*** **<0.01.**

To verify that the observed effect of the drugs affects PrP at the protein level, we next analyzed the expression levels of *PrP* mRNA by quantitative (q)PCR. Comparing the level of *PrP* transcripts in flies fed in normal media or media containing the optimum drug combination of 96 µg/ml 17-DMAG and 24 µg/ml dexamethasone, we found a small reduction in PrP levels that was not statistically significant (*p* = 0.13) (Fig. 3C). We further confirmed that the drug combination did not interfere with the UAS/Gal4 transcriptional system by analyzing the expression of an unrelated gene under the control of UAS (GFP). We detected a small change in the expression of GFP in flies fed with the drug cocktail (Fig. 3C), but that difference was not significant either (*p* = 0.13). Thus, feeding flies with the drug combination had no effect on the expression of PrP and GFP, supporting the idea that the drugs target PrP biogenesis and/or stability, but not transcription.

### Combined treatment of 17-DMAG and dexamethasone induces Hsp70 expression

Once we found the combined regimen that induces a significant reduction on PrP levels, we set out to demonstrate that these effects are mediated by Hsp70 overexpression. For this, we used flies generated for the above experiments and quantified the levels of inducible Hsp70 in western blot using a monoclonal antibody that cross-reacts with Hsp70 from many species. The combination of 48 µg/ml of 17-DMAG with 24 µg/ml of dexamethasone produced a 50% increase in the levels of inducible Hsp70, a difference that was statistically significant (*p* = 0.0064) (Figs. 3D, E and Table 4). The combination of 96 µg/ml of 17-DMAG with 24 µg/ml of dexamethasone induced a 60% increase of inducible Hsp70 (*p* = 0.0004) (Fig. 3D and E). A higher concentration of dexamethasone at 48 µg/ml combined with 17-DMAG (48 µg/ml) resulted in a modest increase of inducible Hsp70 (30%) that was still significant (*p* = 0.04). These results support the synergistic activity of 17-DMAG and dexamethasone as Hsp70 inducers, which is ultimately responsible for the reduction in PrP levels.

**Table 4:** Statistical comparisons (ANOVA) for Hsp70 protein levels in flies treated with combinations of 17 DMAG and dexamethasone.

Test details	Mean 1	Mean 2	Mean Diff.	P value Bonferroni's test	P value Dunnett's test	Difference/significant
48+24 v non-treatment	1.403	1	0.4029	0.0072	0.0064	**
96+24 v non-treatment	1.586	1	0.5862	0.0004	0.0004	***
48+48 v non-treatment	1.304	1	0.3044	0.0405	0.0344	*

* ***p*** **<0.05;**

** ***p*** **<0.01;**

*** ***p*** **<0.001.**

To further support the activity of 17-DMAG and dexamethasone as transcriptional inducers of Hsp70, we examined the expression of inducible Hsp70 transcripts by quantitative PCR. For this, we only studied the optimal combination of 17-DMAG and dexamethasone, 96 µg/ml and 24 µg/ml, respectively. Using a set of primers that amplify all the *Hsp70* isoforms, we found that *Hsp70* transcripts increased four-fold in the flies exposed to the drug cocktail (Fig. 3F). This induction of *Hsp70* was similar in flies expressing either GFP (*p*<0.001) or PrP (*p*<0.001) (Fig. 3F), indicating that the effect of the drug cocktail is independent of the accumulation of misfolded, toxic proteins. This is a remarkable induction that supports the increase of Hsp70 observed in western blot.

Since the drug cocktail induced a robust transcription of Hsp70, a control to demonstrate the specificity of this response is Heat shock protein cognate 70-4 (Hsc70-4), the most abundant of the six constitutive isoforms of Hsp70, which do not respond to cellular stress. We analyzed the expression of *Hsc70-4* by qPCR in flies reared in normal media or media containing the optimum drug combination and found no change in mRNA levels (Fig. 3G). We also analyzed the expression of other inducible chaperone that could be transcriptionally induced by the elevated activity of HSF1. Hsp27 is a small chaperone that binds misfolded substrates and prevents their aggregation until larger chaperones can promote their proper folding. In our experiments, the expression of Hsp27 was not affected in flies fed with normal media or media containing the optimum drug combination (Fig. 3G). Thus, these results suggested that the combination of 17-DMAG and dexamethasone have a robust effect on Hsp70, but not on constitutive Hsc4 and Hsp27.

### 17-DMAG and dexamethasone inhibit the formation of pathogenic isoforms

**Figure 4:**
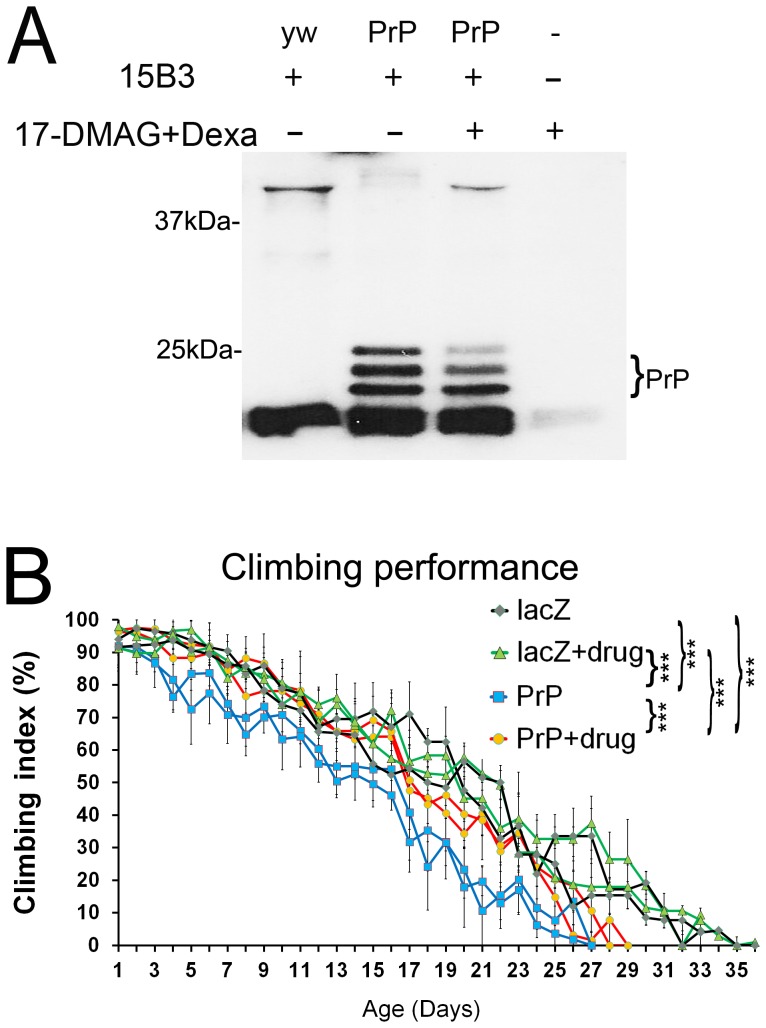
Co-treatment of 17-DMAG and dexamethasone perturbs the accumulation of pathogenic PrP isoforms and improves locomotor dysfunction. **A**, Flies expressing PrP ubiquitously (da>PrP-M6) were fed with 17-DMAG (96 µg/ml) and dexamethasone (24 µg/ml) during development and aged with the drugs for 30 days. Then, we subjected 30 day-old flies to immunoprecipitation with the 15B3 antibody and revealed PrP levels by western blotting. A control experiment with non-PrP flies (yw, lane 1) yielded only non-specific signal from antibody fragments. Flies expressing PrP accumulated 15B3-positive isoforms (lane 2), but when treated with the drug cocktail, less PrP was recognized by 15B3 (lane 3). A control experiment without fly homogenate or 15B3 produced no signal (lane 4). **B**, Climbing ability of adult females expressing LacZ or PrP-M9 in motor neurons with or without the drug cocktail. Non-treated flies expressing PrP in motor neurons (blue lines) induced locomotor dysfunction compared to LacZ flies (black line). PrP flies treated with the drugs (red line) performed significantly better than the non-treated flies. Statistical significance was analyzed by ANOVA multiple comparisons: ** *p*<0.01; *** *p*<0.001.

The reduction in total PrP is relevant from a disease perspective because natively folded PrP (PrP^C^) is the substrate for the formation of pathogenic PrP conformations. Accordingly, we next examined the effect of 17-DMAG and dexamethasone on the accumulation of pathogenic PrP by immunoprecipitation with the 15B3 antibody, a tool developed to recognize PrP^Sc^-like conformations [29]. 15B3 specifically binds misfolded PrP conformations in brain extracts from human and animal prion diseases, but does not bind PrP from healthy subjects. Thus, this is one of the earliest and best-known conformational tools to study PrP. We have shown before that hamster PrP acquires 15B3-positive, PrP^Sc^-like conformations in 30 day-old flies [5]. These pathogenic conformations recognized by the PrP^Sc^-specific antibody 15B3 are PK sensitive, thus sharing properties with proposed pathogenic agents in prion diseases distinct from the PK-resistant infectious agent [30], [31]. To analyze the accumulation of 15B3-positive conformations of PrP, we generated flies expressing a stronger PrP line ubiquitously and aged the flies for 30 days. These flies fed on media containing optimal doses of 17-DMAG (96 µg/ml) and dexamethasone (24 µg/ml) during development and as adults for 30 days post-eclosion. Then, we collected the 30 day-old flies, performed 15B3 immunoprecipitation, and detected 15B3-positive PrP in western blot. As expected, control flies not expressing PrP only produced non-specific signal (Fig. 4A, lane 1), whereas a control experiment without tissue and 15B3 produced no signal (lane 4). Non-treated flies expressing PrP and aged for 30 days cross-reacted with 15B3 (Fig. 4A, lane 2). The three glycoforms resolved neatly, showing lower levels of the diglycosylated isoform. Flies expressing PrP and fed with the 17-DMAG and dexamethasone cocktail also produced 15B3 immunoreaction, but the amount of PrP in each glycoform was significantly reduced, especially the faint diglycosylated band (Fig. 4, lane 3). Thus, the reduction of total PrP affected the dynamics of PrP misfolding and the accumulation of pathogenic conformations.

### 17-DMAG and dexamethasone improve locomotor dysfunction

The results obtained support, so far, the ability of the drug cocktail to promote Hsp70 expression, reduce the steady-state levels of PrP, and reduce the levels of pathogenic PrP conformations in older flies. These observations strongly support the protective activity of the combined treatment with 17-DMAG and dexamethasone. To determine the benefits of the cocktail on the behavioral output of the flies, we examined the locomotor activity. For this, we expressed LacZ (control) or PrP in the motor neurons of adult females with or without the optimum drug combination in duplicates. Then, we tested locomotor performance every day by climbing assay, which takes advantage of the negative geotaxis of flies [32], until all the flies in the group stopped climbing. For these experiments, we used the moderate PrP line because it results in a slow locomotor decline and provides a sensitive assay for testing pharmacologic interventions (Fig. 4B and Table 5). PrP expression resulted in significant locomotor dysfunction (50% climbing by day 15) compared to control LacZ flies (50% climbing by day 20) (*p* = 0.002). The drug cocktail had no effect on LacZ flies, which performed the same as the non-treated LacZ. However, the drugs partially improved the performance of the flies expressing PrP (50% climbing by day 17) compared to the non-treated flies (*p*<0.0001). Although the difference at the 50% climbing point was small, the performance with the drugs was consistently better through the whole assay and consistent in the duplicates, thus explaining the statistical difference (Table 5). Hence, this experiment supports the behavioral benefits of treating flies expressing PrP with 17-DMAG and dexamethasone.

**Table 5:** Statistical comparisons (ANOVA) for climbing activity.

Test details	Mean 1	Mean 2	Mean Diff.	P value Bonferroni's test	P value Tukey's test	Difference/significant
PrP v PrP+drugs	36.28	45.5	−9.22	<0.0001	<0.0001	***
LacZ+drugs v PrP+drugs	51.31	45.5	5.808	<0.0001	<0.0001	***
LacZ v PrP+drugs	50.14	45.5	4.639	<0.0001	<0.0001	***
LacZ+drugs v PrP	51.31	36.28	15.03	<0.0001	<0.0001	***
LacZ v PrP	50.14	36.28	13.86	<0.0001	<0.0001	***
LacZ v LacZ+drugs	50.14	51.31	−1.169	0.6164	0.3553	NS

*** ***p*** **<0.001.**

## Discussion

A large number of neurological disorders are triggered by misfolded proteins and, thus, similar pathogenic mechanisms are proposed to underlie neuronal degeneration [33]. Therefore, it is not surprising that Hsp70 misexpression demonstrates a potent protective activity against intracellular amyloids in several animal models, including polyglutamines and α-synuclein by promoting the solubility an degradation of misfolded conformers [34]–[36]. Interestingly, Hsp70 misexpression is also protective against extracellular amyloids, including the amyloid-ß peptide and PrP [5], [37], although the mechanisms mediating this protection are unclear at this time. Therefore, Hsp70 seems a prime therapeutic target against many of these related proteinopathies. In fact, a few Hsp90 inhibitors, which promote HSF1 activity and induce Hsp70 expression, exert neuroprotection in several animal models of amyloids, including polyglutamines [20], [21], [24], Parkinson's disease [18], [38], [39], and cellular models of Alzheimer's disease [40]–[42]. Moreover, HSF1 knockout accelerates the progression of prion disease in mice, further supporting its protective role against pathogenic PrP [43]. These observations make HSF1 a relevant therapeutic target for a diverse group of proteinopathies [44].

Despite these encouraging results, the translation of Hsp70-activating drugs to the clinic has been slow. One of the main problems is the high toxicity and low solubility of geldanamycin and its derivatives 17-AAG and 17-DMAG, making them inadequate for chronic human use. Currently, only one geldanamycin analogue is currently in ongoing clinical trials (phase 3), retaspimycin [IPI-504], for its anti-oncogenic activity [25]. It is not clear whether this family of Hsp90 inhibitors will be useful for chronic application due to the inherent toxicity of blocking this critical protective molecule. Thus, other classes of HSF1 activators and their combination with Hsp90 inhibitors at safer dosages can be useful in the clinical treatment of chronic conditions.

We show here evidence of the synergistic effect of the 17-DMAG and dexamethasone cocktail on PrP and inducible Hsp70. The combined treatment of 17-DMAG and dexamethasone reduced PrP by 50% and doubled the protein levels of inducible Hsp70. This drug cocktail resulted in lower levels of pathogenic PrP conformers and improved locomotor performance. These results are consistent with previous observations showing that even modest increases in Hsp70 can result in strong protective effects [18]. Based on the literature, 17-DMAG and dexamethasone induce Hsp70 by distinct mechanisms: 17-DMAG inhibits Hsp90 and releases bound HSF1, whereas dexamethasone activation of the glucocorticoid receptor stabilizes HSF1 binding to the Hsp70 promoter. The combination of increased free HSF1 and more stable binding to the Hsp70 promoter likely explains the four-fold induction of Hsp70 transcription. However, it is unclear whether higher concentrations of 17-DMAG and higher levels of free HSF1 can continue to further promote Hsp70 expression without the stable binding to the promoter induced by dexamethasone.

Although we propose a likely mechanism to explain the synergistic effect of the 17-DMAG and dexamethasone cocktail (increased transcriptional activation of Hsp70 by HSF1), we cannot rule out other mechanisms. One possibility is that the increased levels of free HSF1, a master regulator of stress proteins, may stimulate the expression of other chaperones. Although we did not detect a significant increase in Hsp27, other chaperones may be activated or small increases in several chaperones may have strong protective effects [19]. Elevated Hsp70 can be protective by promoting PrP degradation, but Hsp70 can have many other protective cellular activities, including intracellular and axonal transport, synaptic activity, blocking apoptosis, and reducing oxidative stress [11]. In addition to HSP induction, other protective mechanisms are also possible since dexamethasone has many documented activities. One relevant activity is the promotion of catabolic processes by the activation of multiple components of the ubiquitin proteasome complex, including ubiquitin and several subunits of the proteasome complex [45], [46]. Given the anti-inflammatory activity of dexamethasone, it is likely that modulation of other protective pathways may contribute to the synergistic activity in combination with 17-DMAG. Other studies have confirmed the interaction of the glucocorticoid receptor with HSF1, but have suggested an inhibitory activity of the glucocorticoid receptor on HSF1 [47]. It is, thus, important to consider that some of the responses to dexamethasone could be cell type-specific and differ upon cellular stress conditions.

While it may be difficult to precisely determine all the cellular effects of this drug cocktail, it is important to emphasize the new, synergistic activities described here against PrP-dependent pathogenesis. This strategy of combining two drugs with different primary targets should be easily translated to the clinic since dexamethasone is FDA-approved and several Hsp90 inhibitors are under consideration for their anti-cancerous activity. Unfortunately, 17-DMAG was discontinued after phase 1 trials due to animal toxicity and 17-AAG after phase 2 due to poor bioavailability. But other Hsp90 inhibitors are still under study, including the geldanamycin analogue retaspimycin (IPI-504, phase 1–3), resorcinol derivatives (phase 1–3), purine analogues (phase 1–2), and derivatives of the C-terminal inhibitor gedunin (toxic in cell culture) [25], [44]. A few HSF1 activators that work independently of Hsp90, like the natural antioxidant celastrol, the amyotrophic lateral sclerosis drug riluzole, and the antiulcer drug geranylreranylacetone, are less characterized mechanistically, but may be useful in the future for combinatorial regimens [44].

Overall, our findings of the neuroprotective activity of the 17-DMAG and dexamethasone cocktail are significant for prion diseases since there are currently no cure or palliative treatments for these aggressive disorders. In fact, no mechanism linked to PrP pathogenesis is at present a target for clinical studies. The only promising therapies in prion diseases are based on passive immunization due to the accessibility of membrane-bound PrP to exogenously administered antibodies against the unstructured N-terminal domain. Interestingly, some C-terminal anti-PrP antibodies show high toxicity in mice due to topological perturbations of PrP and N-terminal antibodies suppress this toxicity [48]. However, passive immunotherapy protocols are still under development to achieve higher antibody titer into the brain and to specifically target pathogenic conformations. We propose here the use of combinational therapies as an initial step to slow the progression of brain pathology. Since the target of this therapy is relatively well understood, it can be combined with anti-PrP antibodies to maximize the effectiveness of each intervention. However, these and other therapies will not exploit their full potential until we develop more sensitive methods to detect at risk patients many years before they exhibit clinical signs – at least a decade. Only then, we will have real a opportunity to test the disease-modifying potential of these anti-PrP therapies and other related proteinopathies.

## Methods

### Drosophila stocks and genetics

The moderate UAS-HaPrP-M9 and strong UAS-HaPrP-M6 lines expressing hamster PrP and the motor neuron (BG380-Gal4) driver were described previously [5]. The reporter strains UAS-LacZ and UAS-CD8-GDP, and the ubiquitous da-Gal4 driver were obtained from the Bloomington Drosophila Stock Center. For expression of the PrP constructs, homozygous females for the Gal4 drivers were crossed with males bearing HaPrP-M9, HaPrP-M6, or LacZ transgenes. The crosses and their respective progenies were cultured at 25°C unless otherwise indicated.

### Pharmacological treatment

17-DMAG was purchased from InvivoGen (cat# ant-dgl-5) and dexamethasone was obtained from Sigma (cat# D2915). Working stock solutions of both compounds were prepared in MilliQ water at 10 mg/ml, aliquoted (10 µl/tube), and stored at −20°C until further use. 17-DMAG was diluted into final concentrations of 12, 24, 48, and 96 µg/ml and dexamethasone into final concentrations of 6, 12, 24, and 48 µg/ml. Drugs were added to 1 mL of JazzMix food, previously melted by microwave and cooled to 55°C, and mixed by vortexing. Similarly, All four concentrations of 17-DMAG and dexamethasone were used to test the effect on PrP levels, but only the combination of 96 µg/ml 17-DMAG and 24 µg/ml dexamethasone was used in 15B3 immunoprecipitation and climbing assays. For biochemical analysis of PrP, the parental cross was kept in 15 mL conical tubes containing 1 mL of standard JazzMix food with or without drugs, and flies from the progeny were collected after eclosion (day 1). For climbing assays, the parental cross was kept in culture bottles for 3 days and 200 early-stage larvae were transferred to 30×95 mm ‘wide’ plastic vials containing 6 mL of JazzMix with or without the selected combination of drugs. After eclosion, flies were aged in 25×95 mm ‘narrow’ vials with 2 ml of food with fresh drugs changed every other day until the end of the climbing assays.

### Tissue homogenate and western blot

For protein analysis, 1–3 whole flies expressing PrP under the control of the ubiquitous da-Gal4 driver were homogenized in 30 µl of RIPA buffer supplemented with Complete Protease Inhibitors (Roche Applied Science). Following homogenization with a motorized pestle, protein extracts were resolved in 12% NuPAGE Bis-Tris gels (Novex) under reducing conditions, electroblotted into nitrocellulose membranes, and probed against anti-PrP 6D11 (1∶10,000, Covance), anti-inducible Hsp70 (1∶1000; ab47455, Abcam), and α-tubulin (1∶200,000, Sigma) antibodies. After incubation with secondary anti-mouse HRP antibodies (1∶2,000, Sigma), membranes were developed with SuperSignal West Pico Chemiluminescent Substrate (Thermo Scientific). Quantification of relative expression in western blot was done from three independent experiments using α-tubulin as loading control for normalization. Statistical differences were determined by ANOVA and multiple comparisons by Bonferroni and Dunnett's methods using the free online version of GraphPad Prism 6. Averages and standard deviation were plotted in Excel.

### Quantitative RT-PCR

To quantify the levels of mRNA in response to drug treatment, 1 day-old flies expressing PrP or CD8-GFP under the control of da-Gal4 were subjected to real-time RT-PCR assays. First, total RNA was isolated from 10 flies (RNAqueous, Ambion) followed by elimination of DNA traces with Turbo DNAse (Ambion) and reverse transcription with the SuperScript III First-Strand Synthesis System (Invitrogen). Then, quantification of mRNA was performed using the StepOne Plus Real-Time PCR System and the SYBR Green PCR Master Mix from Applied Biosystems. *Rp49* (*ribosomal protein 49*) was used as a housekeeping gene for standardizing mRNA expression. Amplification reaction (25 µl) contained 20 ng of cDNA, 12.5 µl of SYBR Green master mix, and 100 nmol of DNA primers. Amplification primers for *HaPrP*: 5′-TTCACGGAGACCGACATCAA-3′ and 5′-ACTCCTTCTGATACTGGGTGGTACA-3′; for CD8-GFP: 5′-TCAGTTCTGTCGTGCCAGTC-3′ and 5′-ATCACAGGCGAAGTCCAATC-3′; for pan-*Hsp70*: 5′-AGCCGTGCCAGGTTTG-3′ and 5′-CGTTCGCCCTCATACA-3′ (described in [49]); for *Hsc70-4*: 5′-AGCGTTCGACCAACAAGG-3′ and 5′-TACGACTCGAGGCCGTTC-3′; for *Hsp27*: 5′-AAAGATGGCTTCCAGGTGTG-3′ and 5′-CCCTTGGGCAGGGTATACTT-3′, and for *rp49*: 5′-AGCACTTCATCCGCCACC-3′ and 5′-ATCTCGCCGCAGTAAACG-3′. Thermal cycling conditions consisted of an initial denaturation step at 95°C for 10 min, followed by 40 cycles of 15 s at 95°C and 1 min at 60°C. All PCR reactions were run in triplicate. The levels of RNA were calculated using StepOne 2.2 (Applied Biosystems), which relies on the comparative Ct method of quantification. Total mRNA levels were normalized to *rp49* and plotted in Excel as average and standard deviation.

### 15B3 immunoprecipitation assay

To detect PrP^Sc^-specific conformations, flies expressing PrP-M6 under the control of da-Gal4 were collected at day 30 and subjected to immunoprecipitation with the 15B3 conformational antibody following the manufacturer's recommendations (Prionics AG). Briefly, single fly extracts were prepared in 40 µl of 15B3 homogenization buffer and mixed with 450 µl of 15B3 IP buffer and 10 µl of rat anti-mouse IgM Dynabeads (Invitrogen) coated with mAb 15B3. After 2 h of incubation at 25°C, we washed the dynabeads and boiled them in loading buffer. Immunoprecipitated proteins were detected by western blot using the 6D11 antibody. Extracts from non-transgenic *yw* flies were used as negative control.

### Locomotor assay

To assess locomotor function, flies carrying PrP-M9 and control LacZ transgenes were crossed with the motor neuron-specific driver BG380-Gal4 at 25°C. Then, larvae were divided into treated and non-treated groups. The non-treated group was raised in standard food at 26.5°C, whereas the treated group was raised in food supplemented with 17-DMAG at 96 µg/ml and dexamethasone at 24 µg/ml. The progeny was collected in 24 h intervals for duplicates and subjected to climbing assays as previously described [5]. Briefly, 20 newborn adult females were placed in empty vials and forced to the bottom by firmly tapping against the surface. After 10 seconds, the number of flies that climb above 5 cm was recorded. This was repeated 8 times every day for 37 days and climbing ability was plotted as a function of age. Climbing series were subjected to ANOVA followed by the Bonferroni's and Tukey's multiple comparisons for differences in activity levels. Averages and standard deviation were plotted in Excel and all the curves were added together on the same template for side-by-side comparison.
